# What's on the Menu? Policies to Reduce Young People's Sugar Consumption†

**DOI:** 10.1111/1475-5890.12194

**Published:** 2020-05-21

**Authors:** Rachel Griffith, Martin O'Connell, Kate Smith, Rebekah Stroud

**Affiliations:** ^1^ Institute for Fiscal Studies University of Manchester; ^2^ Institute for Fiscal Studies; ^3^ Institute for Fiscal Studies University College London; ^4^ Institute for Fiscal Studies University College London

**Keywords:** sugar consumption, sugar‐sweetened beverage tax, advertising restrictions, childhood obesity, D12, H23, I18

## Abstract

Young people in the UK consume far above the maximum recommended levels of added sugar. It is likely that neither they nor their parents fully take account of the future health, social and economic costs of this high sugar consumption. This provides a rationale for policy intervention. The majority of young people's added sugar consumption occurs in the home, where purchases are typically made by parents. This means that understanding the purchase decisions of adults is important for policy design, even if the policies aim to reduce the consumption of young people. We discuss the merits of popular policies, including taxes, advertising restrictions and restrictions on the availability of specific foods, and we identify promising avenues for future research.

## I. Introduction

There is widespread concern about high levels of added sugar consumption, primarily because of its association with obesity and the increased risk of non‐communicable diseases.^1^ In the UK, the government has set the ambitious target of reducing added sugar consumption by 20 per cent.^2^ This is based on research that links sugar consumption to a range of negative health outcomes, including diabetes, dental disease and higher levels of obesity.^3^ Many countries and regions have introduced policies aimed at reducing sugar consumption, including taxes, restrictions on advertising and regulation of the availability of some foods and drinks.

In this paper, we do three things. First, we outline the rationale for policy intervention, which is based on the presence of market failures that lead to excess consumption. Second, we describe patterns of added sugar consumption by young people in the UK. We emphasise that the bulk of young people's consumption occurs in the home – this suggests that adults (parents) play an important role in the purchase decisions that are a precursor to consumption. Third, we discuss some of the most commonly adopted policies and the existing evidence on how effective and well targeted they are. We conclude by discussing some potentially promising areas for future research.

**Figure 1:**
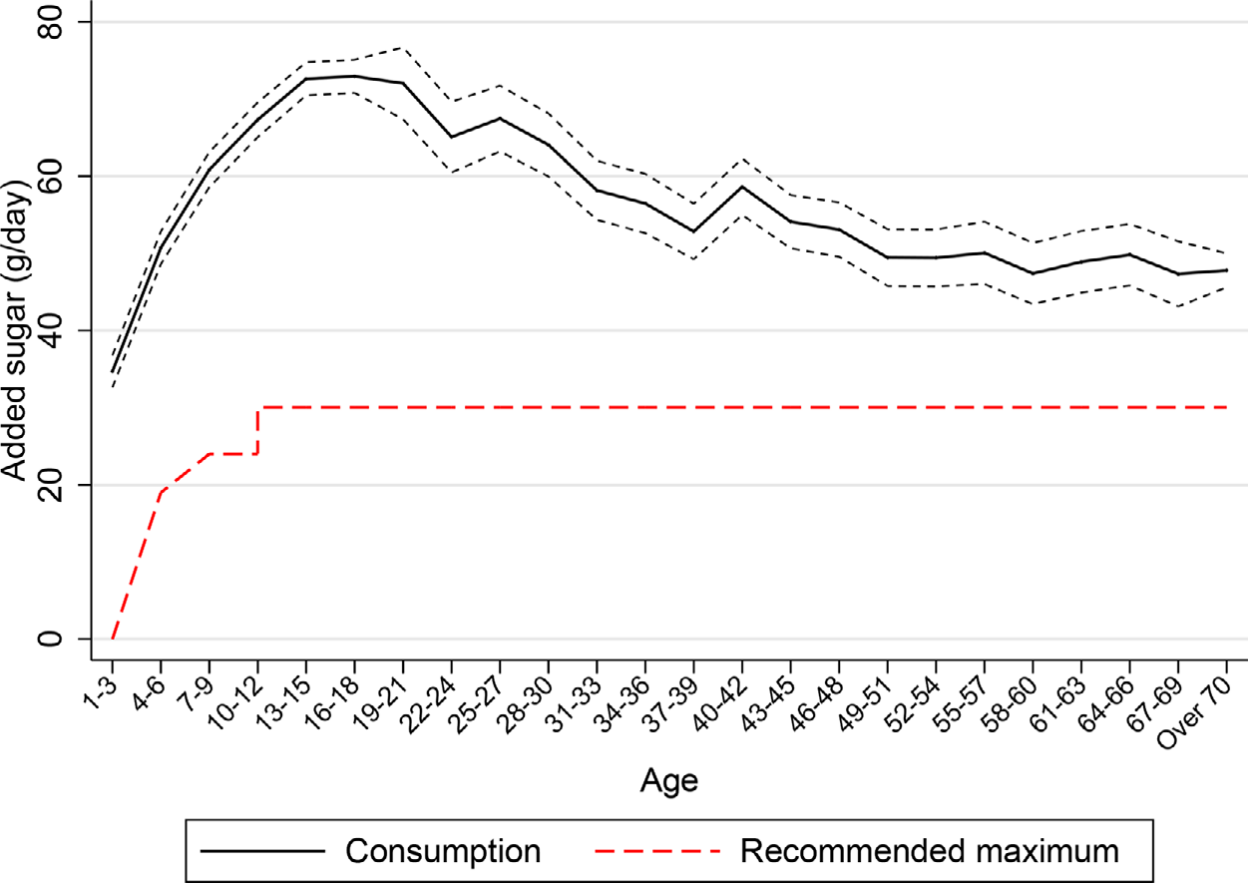
Average added sugar consumption by age *Note*: The solid line shows average daily added sugar consumption (in grams) of individuals in each three‐year age band, based on the National Diet and Nutrition Survey (NDNS; see Appendix A online). Added sugar refers to non‐milk extrinsic sugars, as recorded in the NDNS. The dotted lines show the 95 per cent confidence intervals. The dashed line shows the daily recommended intake of free sugars, as described by the NHS's Eat Well guide (National Health Service, 2017). There are no official guidelines for added sugar consumption for those aged under 4 but it is recommended that they avoid all added sugar consumption (represented by the recommended maximum being shown to be zero for those aged 1–3 in this figure) – see Appendix B online.

Consumption of added sugar^4^ in the UK is well above medically recommended maximum levels, with children and adolescents having particularly high consumption. Figure 1 shows self‐reported consumption (intake) of added sugar from a national representative dietary survey^5^ by age. Young children consume far in excess of the recommended maximum levels. Added sugar consumption is, on average, high in young children (above the maximum recommended for adults) and rises sharply to over 70 grams (g) per day by late adolescence. This is more than twice as much as the recommended maximum of 30g. It gradually declines from around age 21 to an average of around 50g by age 50.^6^

In this paper, we focus on the consumption patterns for young people aged 1–21. This is the group for whom consumption is highest and, as we discuss below, that has been the focus of policy concern. In Section II, we discuss the rationales for public policy intervention aimed at lowering the added sugar consumption of young people and summarise some of the key features of policy design that influence how effective different policies are. In Section III, we describe patterns of added sugar consumption in more detail and discuss the main sources of added sugar for young people and the locations (in the home, at school, at work, on the go) where sugar is consumed. We highlight that the contribution that drinks (and soft drinks in particular) make to dietary added sugar is large and increases in adolescence. We show that the majority of added sugar consumption for those aged 21 or under occurs in the home, which means that parental choices over what food and drink to buy for the household are an important determinant of young people's nutritional outcomes. In Section IV, we discuss some popular policies that aim to reduce excess sugar consumption, particularly among children, including taxes on sugar‐sweetened beverages, restrictions to advertising and regulation of access to sugary foods and drinks. In a final section, we conclude and outline several potentially promising areas for future work.

## II. The policy challenge

The rationale for policy intervention to curb added sugar consumption rests on the existence of costs associated with consumption that are not taken into account at the point of consumption. These might include costs imposed on others (referred to as ‘externalities’) and costs that people impose on themselves in the future (often referred to as ‘internalities’). For brevity, when referring to externalities and internalities collectively, we refer to them as ‘social costs’.

### 1. The existence of social costs

Externalities arise from consumption of sugar if a high level of sugar intake leads some people to have poor health and this imposes costs more broadly on society. For instance, a high‐sugar diet raises the probability that an individual will develop type II diabetes, and this in turn raises the likelihood that they will draw on publicly funded healthcare resources (or increase insurance premiums for other individuals in insurance‐based systems). It is well established that excess sugar consumption is linked with obesity and a range of diet‐related diseases, including diabetes, cancers and heart disease.^7^ The treatment of these diseases is costly – for example, Public Health England (2017a) estimates that the direct cost of treating overweight‐ and obesity‐related illness in 2014–15 was £6.1 billion and that the overall cost to wider society was £27 billion. However, some of these costs might not be externalities, in the sense that they are anticipated and borne by the individual themselves – for example, reductions in productivity will at least partly be reflected in lower wages. There is also a lot of uncertainty about the magnitude of these estimates,^8^ and developing a better understanding of how the external costs vary with the amount of consumption will be an important input to better designing policy and is an area where more research is needed.

In addition to costs borne by others in society, a large part of the future health costs associated with high sugar consumption will be borne by the individual themselves. A number of papers in the medical and public health literature attempt to quantify reductions in the prevalence of various health conditions associated with lower consumption of sugar‐sweetened beverages;^9^ but it is difficult to know the extent to which these costs are ‘internalised’. If these private costs are fully anticipated and understood by the individual (or by a parent on their behalf) at the point of consumption then, however large, they do not justify government intervention. This is because rational, well‐informed consumers will choose an unhealthy diet if the costs of doing so are outweighed by the enjoyment they get from that diet. However, the theoretical literature posits that not all individuals fully account for future costs of consumption.^10^ Unanticipated private costs of this sort are often referred to as ‘internalities’ and arise if people are not fully informed about the health consequences of high sugar consumption, or if they suffer from behavioural biases that mean they make decisions that are not in their long‐term interest.

A prominent example of such behavioural biases is self‐control problems:^11^ an individual values utility today more than utility tomorrow (or in the near future), but values utility at two future dates – say, tomorrow and the following day – roughly the same, and yet, when tomorrow arrives, they revert to valuing the present day more than the following day. Time inconsistencies of this sort can lead to individuals making choices that they later regret. Internalities can also arise when exposure to advertising leads individuals to make decisions that are not in their underlying interest^12^ or, under certain circumstances,^13^ when consumption involves addictive goods.^14^

Many people question whether internalities exist (by their nature, they are difficult to observe) and, if they do, whether they provide a basis for intervention. It is an open question whether or not policymakers really have a better idea of a person's ideal consumption level than the person themselves and, even if they do, it is not clear that it is the appropriate role of government to engage in such paternalistic interventions. The argument that is made in the context of externalities is that intervention is only justified where there are no private solutions, such as bargaining between people.^15^ Analogously, it is argued that private solutions, such as one's own commitment mechanisms – for example, making promises and resolutions and advertising them to those around us – are a better solution to the existence of internalities.^16^

However, in the context of food purchases, both experimental^17^ and circumstantial (through the existence of a multi‐billion‐pound dieting industry^18^) evidence suggests that, despite available commitment mechanisms, people are still making suboptimal choices. In addition, this argument is predicated on the existence of self‐control problems, rather than, say, ignorance of the costs of high‐sugar diets.

### 2. The distribution of marginal social costs

If the presence of non‐trivial social costs provides a rationale for government intervention, then effective policy needs to target those individuals for whom these social costs are highest *at the margin*. This presents a significant challenge in the design of policy, since the size of these marginal costs, and how they vary across different types of people, are difficult to measure. There are two main reasons that we might expect variation in the marginal social cost: (i) convexity in the social costs that are associated with added sugar consumption, i.e. the costs of consuming an extra gram of added sugar is possibly higher for someone who has already consumed a lot of sugar; and (ii) variation across people in the social costs from a given level of added sugar consumption.

Convexity in the relationship between social costs and added sugar consumption might arise if the risk of contracting particular diseases increases non‐linearly with the amount of sugar consumed. For example, at lower levels of sugar consumption, the probability of developing type II diabetes is very small, but this probability may rise non‐linearly in sugar consumption. Hall et al. (2011) show that adults with greater adiposity (more fat) experience larger health gains from a given reduction in energy intake. Measuring the (potentially non‐linear) relationship between social costs and sugar consumption is difficult, and it is also important to recognise that even if there are larger costs for individuals with high‐sugar diets, these need to be unaccounted for at the time of consumption in order to warrant government intervention. Despite the uncertainty, policymakers do tend to target those with high‐sugar diets,^19^ perhaps reflecting an implicit belief that their consumption does generate higher marginal social costs.

It also seems likely that there is variation in the social costs generated by different people, even conditional on the total amount of added sugar they consume. This could be due to variation in individual characteristics such as age or differences in behaviour that could offset or exacerbate social costs. For instance, the social costs of a gram of sugar to someone who regularly runs marathons is likely to be trivial compared with that for someone who is obese and has type II diabetes.

Concerns that young people's consumption is associated with particularly high social costs are one of the key motivations posited for targeting policies at a reduction in their consumption. Young people consume more sugar than adults, on average, which means that if the relationship between social costs and sugar consumption is convex, then they are likely to have higher marginal social costs. However, it may also be the case that they have higher marginal social costs than adults, at any given level of sugar consumption – for example, if an adult and a child consume the same amount of added sugar, the social costs are likely to be higher for the child for physiological reasons and because they have longer to live and so a longer time period over which the costs will accrue. The consequences of poor nutrition early in life are profound, with excess sugar consumption being associated with poor mental health and school performance in children, and poor childhood nutrition being thought to be an important determinant of later‐life health, social and economic outcomes and of persistent inequality.^20^

It is also likely that young people are less inclined to take into account the long‐term consequences of poor dietary choices. For instance, Ameriks et al. (2007) show that the young suffer more from self‐control problems than older people. This is likely to be particularly important if they purchase their own food or are influential in parental purchases, but may also matter if they choose what to consume once the food is available in the household. These factors, along with the fact that young people are often the stated target of policy,^21^ motivate our focus on young people in this paper.

The marginal social costs of sugar consumption might also vary by socio‐economic status of the household. There is some evidence that people with low incomes are more likely to exhibit behaviour that creates internalities.^22^ However, this does not necessarily mean that, *conditional on consumption levels*, the relationship between social costs and added sugar consumption varies with income. Nonetheless, it is possible that higher‐income households make better compensatory investments, which help to offset some of the social costs, so that the marginal social costs are declining with income. Thus variation in both the desire and ability to offset such costs will also affect the magnitude and distribution of social costs.

Allcott, Lockwood and Taubinsky (2019) use information on people's nutrition knowledge and self‐reported self‐control to estimate what their soft drink consumption would be in the absence of behavioural biases, and use this as the basis for a measure of internalities. They find that lower‐income households consume proportionately more sugar‐sweetened beverages due to these behavioural biases than higher‐income households.

Although there is imperfect information about the precise size of marginal social costs and how they vary across people, there is at least suggestive evidence that marginal social costs are likely to be higher for some types of people. Policy should aim to target the consumption that has the highest marginal social costs; we return to this in Section IV. An important area for future research is to better quantify the social costs of sugar consumption and how they vary both within and across individuals.

## III. Young people's added sugar consumption

Figure 1 shows that, on average, young people (1–21 years old) consume far in excess of the recommended maximum level of added sugar. In this section, we describe the distribution of young people's added sugar consumption in more detail. We describe added sugar consumption using self‐reported consumption (intake) data from the National Diet and Nutrition Survey (NDNS).^23^ The NDNS measures consumption rather than purchases. Purchases are a necessary prerequisite for consumption and are often the target of policy, but it is consumption (or intake) that affects health outcomes. This distinction is important because young people often do not purchase the food and drink that they consume (particularly for the very young and when consumption occurs in the home). This means that parental purchase decisions are an important input into young people's consumption decisions and health outcomes.

The NDNS is likely to suffer from under‐reporting,^24^ meaning that added sugar consumption is likely to be even higher than described here. This under‐reporting is not limited to the NDNS, but rather is a feature of intake data more widely.^25^ There is also some concern that under‐reporting may be particularly prevalent among groups whose consumption is associated with high levels of social costs, such as those from households with a lower socio‐economic status, although the evidence on this is mixed.^26^

### 1. Distribution of added sugar consumption

**Figure 2:**
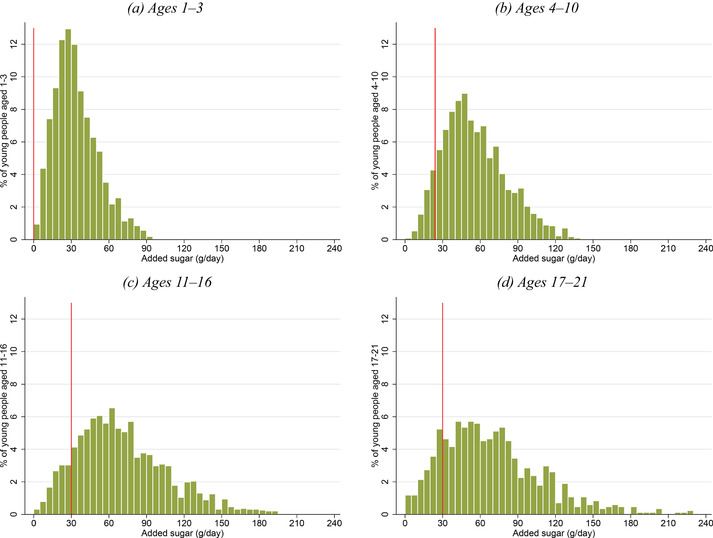
Distribution of added sugar consumption by age *Note*: The graphs show the distribution of added sugar consumption (grams per day) for young people aged (a) 1–3, (b) 4–10, (c) 11–16 and (d) 17–21. Each figure is trimmed at the 99^th^ percentile of the distribution for each age group. Bin width is 5g. The vertical lines represent the recommended maximum daily added sugar consumption for young people in each age range (for ages 4–10, we use the recommendation for those aged 7–10 (24g) rather than for those aged 4–6 (19g)). Data are from the NDNS; the sample comprises 6,112 individuals aged 1–21 over 2008–16. See Appendix A online for more details on the data.

Figure 1 shows the *average* consumption of added sugar at different ages. At all ages, there is also a great deal of variation in how much added sugar young people consume. Figure 2 shows the distributions of added sugar consumption by age groups; the vertical line in each panel shows the recommended maximum added sugar consumption for each age group. Over 80 per cent of those aged 4–21 consume more than 30g per day (the recommended maximum for those aged over 11). Of those aged 11–21, 25 per cent consume more than three times the recommended maximum. If there are convexities in the relationship between social costs and added sugar consumption, then the marginal social costs are likely to be higher for these individuals, and they should thus be the primary target of policy. In older age groups, very high levels of added sugar consumption are less common, as shown in Appendix C online. This provides further motivation for policy's focus on young people's added sugar consumption.

### 2. Sources of added sugar consumption

**Figure 3:**
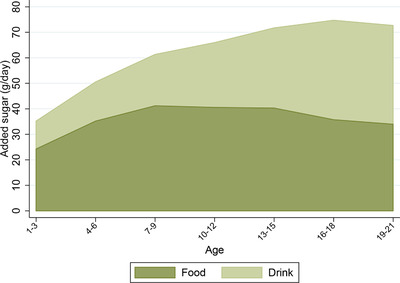
Added sugar consumption from food and drink by age *Note*: The darker area shows the average amount of added sugar (grams per day) that individuals in each three‐year age band get from food, while the lighter area shows the average amount of added sugar (g per day) that they get from drink. Data are from the NDNS; the sample comprises 6,112 individuals aged 1–21 over 2008–16. See Appendix A online for more details on the data.

It is informative for policy design to understand the main sources of young people's added sugar consumption. These indicate the types of food and drink that should be the focus of any policy intervention. Figure 3 shows the amounts of added sugar from food and from drink. The importance of drinks as a source of added sugar increases throughout childhood, accounting for around 30 per cent of added sugar at ages 1–3 and for just over half by late adolescence. On average, added sugar consumption *from drinks alone* exceeds the recommended daily maximum level at almost all ages.

**Figure 4:**
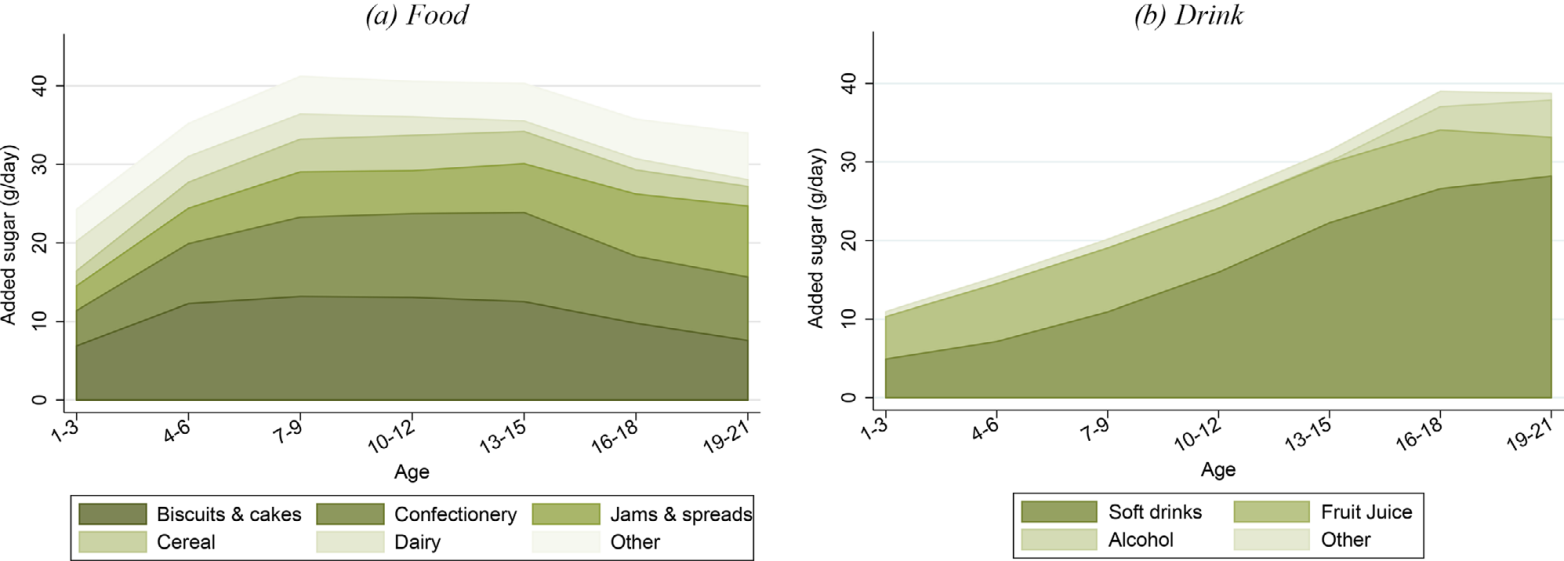
Added sugar consumption by type of food and drink and by age *Note*: The graphs show the average amount of added sugar (grams per day) that individuals within each three‐year age band obtain from each food group (left‐hand panel) and each drink group (right‐hand panel). Data are from the NDNS; sample comprises 6,112 individuals aged 1–21 over 2008–16. Groups are ordered (from bottom to top) in terms of the average amount of added sugar that individuals under 22 years old get from that food or drink group (with the exception of ‘other’, which is placed at the top). ‘Other’ food includes (in order of average contribution to added sugar consumption) fruit and vegetables, miscellaneous, sauces, pasta and rice, meat and fish, toddler food, bread, eggs, crisps and potatoes. ‘Other’ drinks include (in order of average contribution to added sugar consumption) milk and hot beverages. See Appendix A online for more details on the data, including food group definitions.

Figure 4 shows the main categories of food and drink that contribute to added sugar consumption at different ages. Confectionery, biscuits and cakes account for, on average, around half of added sugar consumption from food. Soft drinks are an important source of added sugar for adolescents and young adults: by age 16, the average intake of added sugar from soft drinks almost exceeds the recommended maximum. Over 30 per cent of young people exceed the maximum recommended level of added sugar consumption purely from the added sugar from soft drinks. Fruit juice is also an important contributor to added sugar intake, contributing roughly the same amount of sugar as soft drinks for very young children, aged 1–3. In terms of added sugar, alcohol is as important as fruit juice by age 19, with each making up about 10 per cent of total added sugar consumption.

In Table 1, we describe how the importance of soft drinks, confectionery, and biscuits and cakes (three of the largest sources of young people's added sugar) varies with the total amount of added sugar in young people's diet. This is informative for designing policy if there are convexities in the social costs associated with added sugar consumption that mean that the marginal social cost is higher for those who consume a lot of added sugar.

**Table 1:** Share of added sugar from soft drinks, confectionery, and biscuits and cakes

	*Percentage of added sugar from*:		
*Total added sugar in diet (g/day)*	*Soft drinks*	*Confectionery*	*Biscuits and cakes*
<30	11.2	8.8	21.6
30–60	18.3	12.2	20.7
60–90	25.8	13.4	17.8
>90	36.0	16.5	13.9

*Note*: This table shows the share of added sugar that individuals get from soft drinks, from confectionery and from biscuits and cakes, by the total daily amount of added sugar they consume. Data are from the NDNS; sample comprises 6,112 individuals aged 1–21 over 2008–16. See Appendix A online for more details on the data.

Soft drinks and confectionery together account for over half of the added sugar consumed by individuals with very high added sugar diets, compared with only 20 per cent of the added sugar consumed by individuals who consume less than the maximum recommended daily level of 30g. By contrast, biscuits and cakes contribute a larger share of added sugar to those with low added sugar diets, making up just over 20 per cent of added sugar consumed by individuals who consume less than the maximum recommended daily level of 30g, compared with 14 per cent of the added sugar consumed by those with very high levels of added sugar consumption.

**Figure 5:**
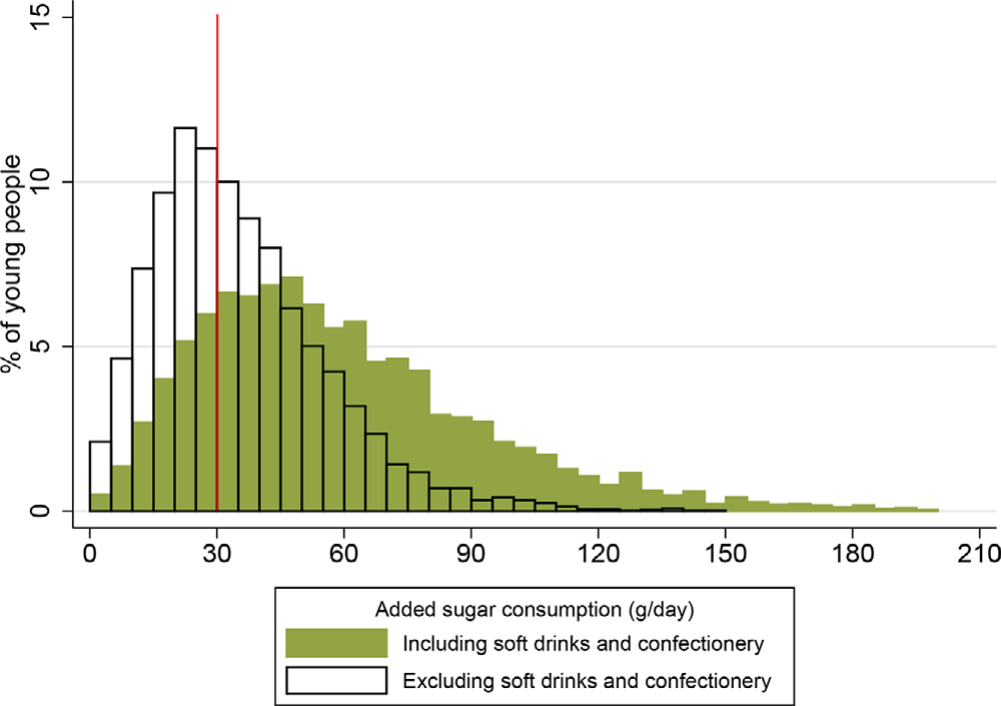
Distribution of added sugar consumption including and excluding sugar from soft drinks and confectionery *Note*: The graph shows the distribution of young people's total added sugar consumption (shaded bars) and added sugar consumption excluding added sugar from soft drinks and confectionery (bars with black outline). The vertical line shows the recommended maximum daily added sugar consumption (30g) for those older than 10 (for young people under 11, the recommended maximum is less than this – see Figure 1). Bin width is 5g. Data are from the NDNS; sample comprises 6,112 individuals aged 1–21 over 2008–16. See Appendix A online for more details on the data.

In summary, these statistics suggest that policies aimed at the confectionery or soft drinks markets will target a substantial portion of the added sugar consumption of young people, particularly for those with diets that are very high in added sugar. However, it should be noted that even if consumption of soft drinks and confectionery fell to *zero*, over 50 per cent of young people would still be consuming more than the recommended daily maximum of 30g of added sugar a day. Figure 5 compares the distribution of the amount of added sugar consumed by young people with how much added sugar they would consume if we exclude soft drinks and confectionery (recommended daily maximum added sugar consumption is shown by the vertical line).

### 3. Who buys the sugar that young people eat?

Many policies that aim to reduce socially excessive sugar *consumption* do so by altering *purchase* incentives – for example, by changing relative prices through taxation, by regulating availability at point of sale or by restricting advertising. In many contexts, the purchase decision is taken by a different person and at a different time from the consumption decision – for example, the main shopper in the household makes purchase decisions over the shopping basket for consumption at home at a future time by other household members.

It is therefore important to understand *who* is purchasing the products that young people go on to consume, and what factors affect the purchaser's decisions, as well as the consumption decision (conditional on purchase). In the NDNS, we do not observe who purchased the food and drink that are consumed. However, we do observe the location in which consumption occurred, which provides an indication of who was the likely purchaser. For example, food and drink consumed at school are more likely to have been purchased by the child, whereas food and drink that are consumed in the home are typically purchased by an adult (parent) household member. This is particularly true when considering consumption by young people under 18, who are less likely to be the main shopper in their household.

A further consideration is whether behavioural biases are more or less likely to be present for particular types of purchase occasion. For example, when an individual is making a purchase decision about food that is for their immediate consumption out of the home, they may be more likely to yield to temptation which they later regret than when they are making a decision over the shopping basket for future consumption. We highlighted in Section II that the marginal social costs of sugar consumption may vary across people; they may also vary across consumption occasions (for a given person) – for example, if a person is more likely to yield to temptation in some situations than in others. If this is so, then policy should aim to target these occasions. However, as with measuring variation in the social costs across people, estimating whether the marginal social costs vary across consumption occasions is challenging.

**Figure 6:**
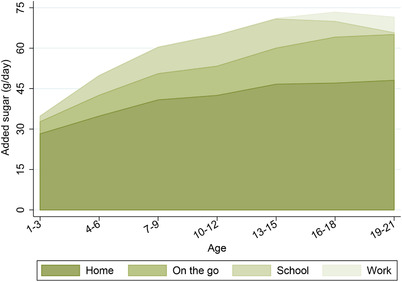
Added sugar consumption by location and age *Note*: The graph shows the average amount of added sugar (grams per day) that individuals consume at home, on the go, at school or at work, as recorded in the NDNS. Sample is 6,112 individuals aged 1–21 over 2008–16. Locations are ordered (from bottom to top) in terms of the average amount of added sugar individuals under 22 years old consume in that location. See Appendix A online for more details on the data.

**Figure 7:**
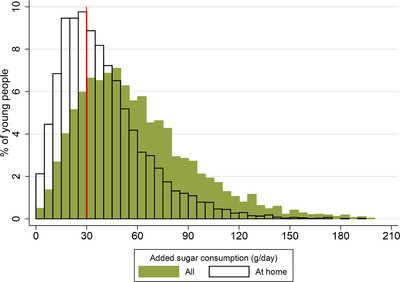
Distribution of total added sugar consumption and at‐home sugar consumption *Note*: The graph shows the distribution of young people's total added sugar consumption (shaded bars) and added sugar consumption at home only (bars with black outline). The vertical line shows the recommended maximum daily added sugar consumption (30g) for those older than 10 (for young people under 11, the recommendation is less than this – see Figure 1). Bin width is 5g. Data are from the NDNS; sample comprises 6,112 individuals aged 1–21 over 2008–16. See Appendix A online for more details on the data.

Figure 6 shows the amount of added sugar that young people consume at home, on the go, at school and at work, on average, for different age groups. At all ages, most added sugar is consumed in the home. However, the share of added sugar that young people consume out of the home is higher than the share of calories they consume out of the home (31 per cent compared with 26 per cent; see Figure B.6 in the online appendix), which reflects the fact that people tend to eat more sugary food outside the home than they do in the home. The share of added sugar consumed at home is particularly large for young children (80 per cent for 1‐ to 3‐year‐olds and 70 per cent for 4‐ to 6‐year‐olds); as individuals get older, a smaller share of added sugar is consumed at home, but it is still in excess of two‐thirds by age 21. This suggests that, even if all the added sugar consumed outside of the home is purchased by the young person, the purchase decisions of parents (or other adults in the household) are still a central determinant of the added sugar that young people consume. Figure 7 compares the distribution of all added sugar consumption with the distribution of added sugar consumed at home. This shows that, even if no sugar were consumed out of the home, almost 60 per cent of young people would consume more than the recommended maximum of 30g of added sugar a day.

**Figure 8:**
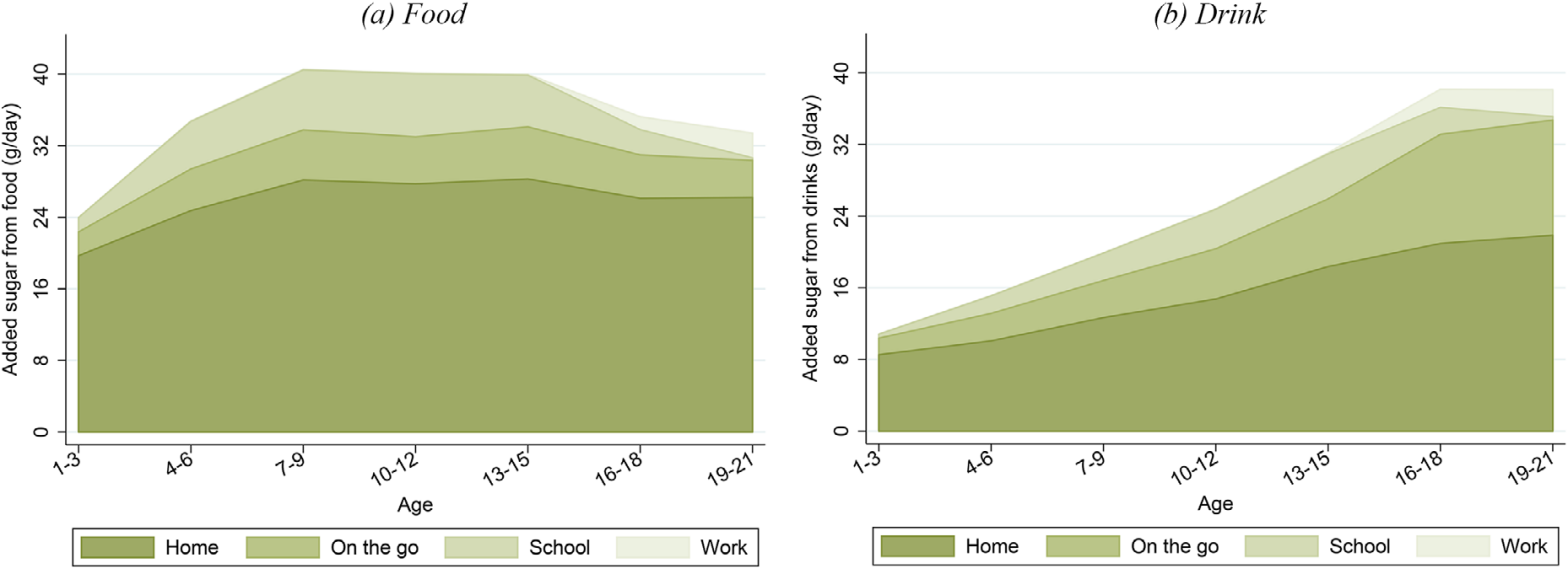
Added sugar consumption from food and drink by location and age *Note*: These graphs show the average amount of added sugar (grams per day) that individuals from each three‐year age band get from food (left‐hand panel) and drink (right‐hand panel) that is consumed at home, on the go, at school and at work. Locations are ordered (from bottom to top) in terms of the average amount of added sugar that individuals aged under 22 years consume in that location. Data are from the NDNS; sample comprises 6,112 individuals aged 1–21 over 2008–16. See Appendix A online for more details on the data.

Figure 8 shows how the location of consumption of added sugar varies for food and drink. The bulk of added sugar from food is consumed in the home, and the share consumed at home is higher for food than for drink. The importance of consumption out of the home for both food and drink increases with age, with the increase more pronounced for drink. The increased importance of added sugar from drink consumed out of the home is entirely driven by increased soft drinks consumption. Although out‐of‐home consumption is increasingly important at older ages, it is still the case that the majority of added sugar from both food and drink is consumed in the home.

In Table 2, we show how consumption outside of the home varies with the total amount of added sugar that young people consume. Individuals with a higher added sugar diet tend to consume more of that added sugar out of the home, and tend to get a relatively high share of the added sugar that they consume from soft drinks and confectionery while out of the home. For instance, for those with very high added sugar diets, more than 45 per cent of added sugar consumption from soft drinks occurs out of the home, compared with less than 30 per cent for those whose added sugar consumption falls below the recommended maximum level of 30g a day. This underlines that, even though home consumption contributes most to added sugar, out‐of‐home consumption is nonetheless important, especially for those with the highest added sugar diets.

**Table 2:** Share of added sugar that is consumed out of the home

*Total added sugar in diet (g/day)*	*Percentage of added sugar from food and drink consumed out of the home*	*Percentage of added sugar from soft drinks consumed out of the home*	*Percentage of added sugar from confectionery consumed out of the home*
<30	23.0	29.3	28.7
30–60	31.2	39.0	37.4
60–90	33.4	43.2	39.8
>90	36.2	45.5	46.0

*Note*: This table shows the share of added sugar that young people get from all food and drink, from soft drinks and from confectionery that they consume out of the home, by the total daily amount of added sugar they consume. Data are from the NDNS; sample comprises 6,112 individuals aged 1–21 over 2008–16. See Appendix A online for more details on the data.

## IV. Policy solutions and their effectiveness

Many governments have introduced policies that aim to reduce sugar consumption, in order to tackle rising childhood obesity and poor nutrition.^27^ In this section, we discuss some of the most popular types of policies, the factors that influence how effective they are, and open questions for future research.

### 1. Taxes on sugar‐sweetened beverages

A number of jurisdictions have adopted taxes on sugar‐sweetened beverages. These taxes are often known as ‘soda taxes’; however, they typically apply to a broader set of products than carbonated soft drinks (or sodas). Mexico and the UK introduced taxes on sugar‐sweetened beverages in 2014 and 2018, respectively. The US cities of Berkeley, San Francisco, Oakland, Albany and Boulder all have taxes on sugar‐sweetened beverages. France and the US city of Philadelphia have taxes that apply both to sugar‐sweetened and diet drinks.

The use of taxes to correct for the existence of externalities associated with consumption dates back to Pigou (1920). More recently, there have been calls for the use of such taxes to also address internalities.^28^ The logic behind the use of such ‘corrective taxes’ is that, by raising the price of the taxed good, they induce consumers to internalise the social costs associated with their consumption. In a stylised model in which the social costs of an extra gram of sugar intake are the same for all individuals (say *X* pence) and the market is perfectly competitive (so, in the absence of a tax, the good is sold at cost), a tax per gram of sugar of *X* pence is fully corrective, meaning that consumers and producers fully internalise the additional social cost associated with consumption. However, as we discuss in Section II, it is likely that the social costs of excess sugar consumption vary across individuals.

When consumption by some groups of the population creates social costs and consumption by another group does not (or creates much smaller costs), then a tax levied on the offending goods is no longer fully corrective (apart from in the unlikely case that the policymaker is able to set tax rates that differ between the two groups of individuals). The optimal rate then trades off the aim of lowering the social costs of consumption of one group, against the desire not to unnecessarily distort the decisions of the other group. Tax design can be improved in this situation by levying higher taxes on types of products that are more popular among the group of people whose consumption generates higher social costs.^29^ For instance, if children only consume fruit squash, then a tax on squash may be more effective at targeting the sugar that they obtain from drinks than a broader sugar‐sweetened beverages tax. This will, however, depend on the extent to which children may switch away from the taxed fruit squash to other sources of sugar.

If consumers are willing to switch away from sugar‐sweetened beverages to other sources of sugar, this will make a tax on sugar‐sweetened beverages less effective at reducing sugar consumption. In most jurisdictions, fruit juice and milkshakes are outside the scope of sugar‐sweetened beverages taxes. Yet these products are both very high in sugar and likely to be relatively close substitutes for the taxed sugar‐sweetened beverage products.^30^ The more that a decline in the consumption of sugar‐sweetened beverages is offset by higher intake of these alternative sugary drinks, the less effective the policy will be in tackling the social costs of sugar consumption. Similar reasoning applies to substitution from sugar‐sweetened beverages towards high‐sugar food products. However, there is some evidence that this might be a less important margin than switching between drinks: experimental evidence suggests that food and drink are not that substitutable – people consume the same amount of food regardless of how much (non‐alcoholic) drink they consume.^31^

An additional complication in the optimal design of such taxes is that many markets for heavily branded food and drink products are highly concentrated, and not perfectly competitive. This means that manufacturers set prices that are higher than the marginal cost of production. In this case, there are two market failures: social costs of consumption and positive price–cost margins. Tax policy may reasonably be targeted at social costs of consumption, but it is less clear it should be used to correct for the presence of positive margins, particularly given that government has other tools at its disposal (such as competition policy) that are likely to be more effective at dealing with imperfect competition. Despite this, it is important to recognise that the response of firms will determine how effective a tax is at reducing sugar consumption. In imperfectly competitive markets, the transmission of the tax into price changes will most likely not be 100 per cent. Taxes can be under‐ or over‐shifted to consumer prices (depending on the shape of demand and the nature of firm competition), and will vary across products, which will have a bearing on the effectiveness of the policy in targeting social costs.^32^

The specific structure of a tax is also an important determinant of its likely effectiveness. Policymakers have usually chosen to levy sugar‐sweetened beverage taxes in volumetric terms (i.e. the tax is per litre of drink, rather than per gram of sugar). Typically, there is a single rate of tax applied to all sugar‐sweetened beverages (and, in a few cases, to diet products too). The UK tax is an exception. It is volumetric; however, it has three bands – a zero tax band for products with less than 5g of sugar per 100ml, a lower rate for products with 5–8g of sugar per 100ml and a higher rate for more sugary products. To the extent that sugar from the products is the source of concern, the tax should be levied directly on sugar content. This both ties the tax more closely to the source of social harm and creates incentives for firms to reformulate their products to lower their sugar content. The banding in the UK's volumetric tax means that this policy is closer to targeting sugar than in other jurisdictions, though it fails to do this as well as a tax that is proportional to sugar content would do.

Corrective taxes in general, and sugar‐sweetened beverages taxes in particular, have attracted criticism from some groups for being regressive.^33^ This point is usually made on the basis that these products tend to be more popular with low‐income consumers, and therefore this group will bear a disproportionate burden of the tax. However, the assessment of regressivity is more subtle in the presence of internalities. If low‐income groups are more likely to suffer internalities (either because of higher sugar consumption or because of lower compensatory investments that offset the bad effects of poor diet), the tax may achieve bigger gains from averted internalities among these groups, potentially overturning the regressivity of the traditional economic burden of taxation.^34^ In addition, the government has other tools to achieve its distributional aims; it therefore matters (from a distributional perspective) how the revenue raised from corrective taxes is distributed, and how other aspects of the tax and benefit system are adjusted in response to the adoption of such taxes.

### 2. Advertising restrictions

In recent years, a number of countries, including the UK, Canada, Chile, Norway and Mexico,^35^ have introduced restrictions on advertising unhealthy foods to children, with the aim of reducing children's sugar consumption. The reasoning that motivates introducing such restrictions is that advertising acts to ‘persuade’ people to make purchase decisions that they otherwise would not make and that are not in their underlying interest.^36^ The extent to which advertising does distort decisions is uncertain;^37^ however, there is a perception that children, in particular, are unable to process the persuasive intent of advertising.^38^ This has meant that advertising restrictions have primarily been targeted at reducing the advertising exposure of children. However, as discussed in Section III, the majority of children's sugar consumption comes from food and drink purchased by adult household members. This means that the advertising exposure of both adults and children is relevant for children's consumption and that advertising restrictions targeting the television that children and adults watch may be an effective policy (even in the absence of advertising distorting people's purchase decisions) if advertising increases consumption that generates externalities.

In the UK, food and drink high in fat, sugar or salt (abbreviated to ‘HFSS’, but to which we refer as ‘less healthy’) cannot be advertised during children's programming, defined as programmes for which children are the target audience or constitute over 25 per cent of the audience.

**Figure 9:**
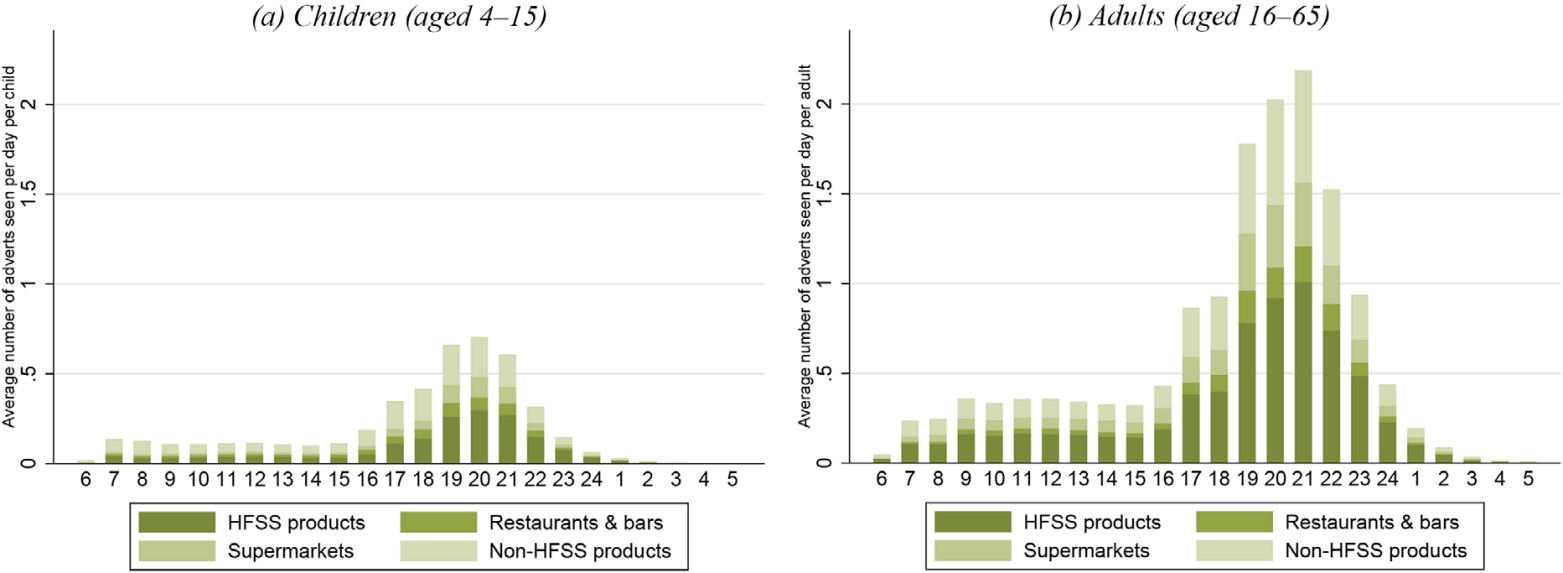
Average number of food and drink adverts seen on television per person, by hour of the day and food type *Note*: Each bar shows the number of adverts seen per child (left‐hand panel) and per adult (right‐hand panel) in each hour of the day, on average, during 2015. Adverts are classified as being for ‘HFSS products’ or ‘non‐HFSS products’ on the basis of nutritional content of products advertised. ‘Restaurants & bars’ are largely fast food restaurants. ‘Supermarkets’ include all advertising carried out by supermarkets where no specific product was advertised. For more details on the advertising data, see Appendix D online.

In Figure 9, we show the average number of adverts seen by children (aged 4–15) and adults (aged 16–65) during each hour of the day for different types of food and drink in 2015 (measured per person per day).^39^ Adults see over double the number of adverts for food and drink that children do. This is partly because adults watch a larger volume of television than children,^40^ but also due to a higher density of food and drink adverts during the television watched by adults. Despite the fact that advertising of less healthy products is banned during children's programming, half of the food and drink advertising that children see on television is for less healthy products or for restaurants and bars (most of which are fast food outlets). This is because much of the television that children watch is not during children's programming.

Concern about the amount of less healthy food and drink advertising to which children are exposed has led to calls to extend advertising restrictions. The UK government is considering whether to extend the ban on advertising less healthy products to include all programmes shown prior to the 9 p.m. watershed.

The extent to which advertising restrictions are successful at reducing children's sugar consumption depends on how effective they are at reducing advertising exposure of both children and their parents, how a reduction in exposure affects purchase decisions and how firms respond by changing other factors that affect consumers’ purchase decisions, such as prices or product characteristics (for example, nutrient content).

Seventy per cent of the advertising for less healthy products that children see, and 60 per cent of the advertising for less healthy products that adults see, is before the 9 p.m. watershed and would be directly impacted by the proposed extended restrictions. Nonetheless, it is unlikely that exposure to advertising for these products would fall by such magnitudes in the event of a ban: firms are likely to shift their advertising when faced with restrictions. There is evidence that following the 2007 ban on advertising less healthy products on children's television, restricted adverts were shifted from children's television to non‐children's television.^41^ In 2015, 95 per cent of the less healthy food and drink brands that were advertised on television were advertised both before and after the watershed, suggesting that many firms are willing to advertise both pre‐ and post‐watershed, even without timing restrictions. Another way firms might respond is to shift adverts to other media, such as online. This is likely to be an increasingly attractive alternative to television advertising given that fewer children and adults are watching live TV than in the past.^42^

The effectiveness of advertising restrictions depends not only on the magnitude of the reduction in exposure that they achieve but also on the degree to which a reduction in exposure achieves a reduction in excess sugar consumption. A key channel is how purchases, a necessary precursor to consumption, are influenced by advertising. Advertising can have both rivalrous and expansionary effects.^43^ Rivalrous advertising leads consumers to switch between rival brands (for example, from Coke to Pepsi), whereas expansionary advertising leads to an increase in purchases in the market as a whole (for example, for all soft drinks). The effects of advertising may well vary across product types and even within markets. Dubois, Griffith and O'Connell (2018) find that brand‐level crisps adverts lead some consumers to switch between rival brands, but also lead to an overall expansion in demand for crisps. If advertising is primarily rivalrous, then restrictions are likely to have a smaller effect on total sugar purchases.

The effect of advertising exposure on sugar purchases also interacts with the design of advertising restrictions and firms’ response to them. For example, UK restrictions apply to products that are high in fat, sugar or salt and to brand advertising that ‘has the effect of promoting an HFSS product’.^44^ This means that firms are free to advertise healthier (i.e. non‐HFSS) products, or their wider brand, which may act to increase demand for similar less healthy products. As an example, McDonald's advertise during children's television by featuring carrots in place of their less healthy products, which may act to stimulate demand for other McDonald's products. If there are brand spillovers to advertising, then firms have an incentive to advertise existing healthier products in the place of their less healthy products, potentially leading to smaller‐than‐anticipated reductions in sugar consumption.

There are other dimensions of firms’ strategic response that will affect the transmission of advertising restrictions through to purchases. Firms may respond to advertising restrictions by altering product characteristics to enable them to continue to advertise their products (i.e. reformulating their product to create a healthier product). This could act to strengthen or weaken the effect of advertising restrictions on sugar purchases. If firms change the nutrient composition of their products, and consumers switch to (or carry on) buying the healthier alternatives, then this could act to reduce sugar purchases. On the other hand, if firms create or reformulate products in order to continue to advertise their broader brand, and advertising of the brand stimulates demand for sugary varieties, then the reduction in sugar purchases could be less than anticipated.

Firms are also likely to adjust prices in the face of constraints on when and how much they can advertise; whether advertising restrictions will lead to price reductions or price rises depends in part on the nature of advertising. If advertising plays the role of informing consumers about prices, then restricted advertising can lead to a lessening of competition in the market, and higher prices. However, if advertising acts to persuade people to purchase a product (without providing them with information about the product's price or quality), then restricting advertising may lead consumers to be less willing to pay for the products, giving firms an incentive to lower their prices.^45^

Children's exposure to advertising of unhealthy foods is often the target of policy. However, as discussed in Section III, the majority of children's sugar consumption comes from food and drink purchased by adult household members. This means that the advertising exposure of both adults and children is relevant for children's consumption and that we need to understand more about how household purchases are allocated between individuals in that household. There is evidence that advertising can lead to increased consumption, conditional on the product being available.^46^ Understanding intra‐household allocation decisions is an active area of economic research,^47^ and a useful direction for future research is to study how the advertising exposure of various household members affects both the purchases made at the household level and how these purchases are split between members of the household when consumed.

### 3. Other policy interventions

There are a range of other policies, in addition to taxes on sugar‐sweetened beverages and advertising restrictions, that have been either introduced or discussed as ways to reduce children's excess consumption of sugar. These include policies that restrict or change choice sets (for example, removing confectionery from schools or by tills in shops, encouraging product reformulation, and introducing planning regulations on the opening of fast food outlets) as well as policies aimed at improving information provision (for example, mandatory labelling of food products).

#### a) Changing choice sets

There are a number of measures in place that seek to alter people's, and particularly children's, choice sets (i.e. the set of products they choose between). Restricting advertising is one of these policies; one effect that advertising has is to make a product more prominent in people's choice sets.^48^ Other measures take the form of government seeking to persuade or apply pressure on the food industry to alter its behaviour, without the introduction of mandatory regulation. For instance, several UK supermarket chains have decided to remove confectionery and other unhealthy snacks from near tills. To the extent that purchases of these products are impulse buys (perhaps due to people failing to exercise self‐control), or purchases that parents make after giving in to pressure applied to them by their children, this change may successfully remove the products from consumer choice sets precisely at the point in time when they are most likely to make an ill‐judged decision. In contrast, people who intend to make planned purchases of the products can still do so by seeking them out in the correct aisle of the store.

Ejlerskov et al. (2018) find evidence that removing less healthy snacks from tills leads to a reduction in purchases of those products. An important area for future research will be to investigate the robustness of these results to other settings, and also to understand heterogeneity in the effects. The impact of such policies on nutrition and health outcomes depends not only on the aggregate reduction in purchases, but also on *who* responds to the policies. The more the reductions are from people whose consumption has high marginal social costs, the more effective the policies will be at tackling the most socially costly forms of consumption.

A related policy idea is to restrict the foods that are available to purchase in schools. Since 2006, foods high in fat, salt or sugar have not been permitted to be sold in UK schools. Similar restrictions have also been introduced in other countries. For example, in 2005, France introduced a ban on vending machines in secondary schools. Capacci, Mazzochi and Shankar (2018) find that the ban led to a 10g reduction in sugar intakes from morning snacks at school, as well as a significant reduction in the frequency of morning snacks. However, they find no evidence that the ban led to a reduction in total daily sugar intake, suggesting that pupils compensated by increasing sugar intake at meals or other points in the day. It is also important to note that, as discussed in Section III, 15 per cent of children's added sugar consumption occurs in schools. This suggests that, even if such policies reduced consumption of added sugar in schools completely, children would still be consuming far more sugar than is recommended.

Another way to change people's consumption is to alter the characteristics of the products that are available to them. Griffith, O'Connell and Smith (2017) study the UK government's salt reduction strategy introduced in 2005. They find that the reduction in salt intensity of grocery purchases was entirely driven by voluntary reformulation targets, and not due to consumer switching. Targeting firms via the introduction of voluntary reformulation targets recognises that consumers may have difficulty understanding nutrition information and may lack the motivation to make use of it. Griffith et al. show that households in the lowest socio‐economic groups purchase the most salt (per person), and the observed decline in the salt intensity of their grocery purchases was entirely due to reformulation.

#### b) Information provision

Another set of policies seek to improve consumers’ knowledge of the benefits of a healthy diet and the nutritional content of the food they buy. Such policies are predicated on the fact that some consumers lack information, and that it is this lack of information (as opposed to, say, cognitive failings in the ability to process or act on the information) that prevents them from making fully informed decisions.

In the UK, policies such as mandatory food labelling are aimed at improving knowledge of the nutritional content of products, and policies such as the five‐a‐day campaign are aimed at improving consumers’ knowledge of what constitutes a healthy diet. Capacci and Mazzocchi (2011) look at the effects of the five‐a‐day information campaign, which aimed to encourage more fruit and vegetable consumption in the UK. They find that, on average, the campaign led to the consumption of an additional 0.3 portions of fruit and vegetables per person per day relative to an average initial consumption of just under four portions.

Nutritional information, even when provided, is not always easy to digest. It is often shown in a numerical format (for example, number of grams of sugar or as a percentage of recommended daily intake) which, particularly at a glance, is not easy for some consumers to understand. For example, Bhutoria, Jerrim and Vignoles (2015) suggest that around one‐third of people could not understand a simple percentage. Some policies aim to improve the ease of interpreting this sort of information. An example of such a policy is the traffic lights food labelling system, introduced in the UK in 2013. The labels consist of a series of green, amber or red coloured labels on the front of a product's packaging, which indicate whether the product has low (green), medium (amber) or high (red) amounts of fat, saturated fat, sugar and salt. In addition to simplifying the amount of information that consumers need to process, the traffic light labels also draw on consumers’ automatic associations between red and ‘stop’ (for less healthy foods) and green and ‘go’ (for healthier foods).^49^ Thorndike et al. (2012) look at the effect of traffic light labelling on purchases in a hospital cafeteria. They find that the introduction of traffic light labelling led to an increase in the sale of healthy foods (labelled green) and a decrease in the sale of unhealthy foods (labelled red). Levy et al. (2012) find that the effect of labelling on purchases is greatest for less educated hospital workers. This suggests that providing easy‐to‐digest nutritional information may particularly improve the decisions of people who have more difficulty processing complex nutritional information. An interesting avenue for future work would be to look at how these effects vary across adults and children. It will also be important to assess the external validity of these findings; one might reasonably think that choices made in hospital or by hospital workers are not necessarily representative of those made by the population as a whole.

It is also important to consider the general equilibrium effects of such policies. Capacci and Mazzocchi (2011) find that the impact of the five‐a‐day campaign was partially offset by an increase in the price of fruit and vegetables. These price rises may have been driven by factors unrelated to the policy, but they could also, in part, reflect sellers raising prices in response to the demand increase stimulated by the policy. If the policy led to a price increase, then some groups (for example, those unresponsive to information but responsive to price changes) could have reduced their consumption following the introduction of the policy.

## V. Final comments

Young people consume far above recommended levels of added sugar and are unlikely to fully take account of the future health, social and economic costs of this excess consumption. This provides a rationale for government intervention. In this paper, we discuss the challenges around designing effective policy that aims at tackling the market failures present in this setting. There are a range of policies that have either been introduced or proposed, and the success of these policies will depend on the nature of the market failure and on how people respond to changes in the economic environment that they face. We show that much of young people's added sugar consumption occurs at home and is likely purchased by their parents. This means that it is important to understand how policies affect parents’ purchase decisions and the degree to which they take into account the long‐term health costs associated with their children's food consumption.

We discuss a number of existing policies and point to areas where there is scope for refining such policies. For example, a sugar‐sweetened beverage tax levied on sugar content, rather than in volumetric terms, would be more effective at correcting for the social costs associated with soft drink consumption. We also point out that advertising regulations that restrict adverts for unhealthy products, but continue to allow firms to advertise similar brands where the effect of advertising spills over onto the unhealthy product, may provide scope for firms to circumvent restrictions. More broadly, policy should be targeted as closely as possible at reducing consumption of the sources of social costs (for example, sugar), and its design should anticipate the likely strategic responses of firms operating in the market.

There is much still to be learned in order to help design effective (or optimal) policy in this setting, which makes this an exciting area for future research. One of the biggest unknowns that limits our ability to make concrete policy recommendations is the magnitude of, and the variation in, the social costs of excess sugar consumption. In Section II, we discuss the broad drivers of externalities and internalities, but highlight how little we know about their precise magnitude. We need to better understand the link between sugar consumption and health outcomes, and the potential costs associated with such outcomes. We also need to understand the extent to which these costs are not fully anticipated or taken into account by consumers (i.e. are *external* at the point of consumption). Finally, it is crucial to recognise, and have a better understanding of, how these costs vary across people, including by age, existing health conditions and socio‐economic background.

Another important input into designing better policy is understanding market responses to policy interventions. This requires us to know how consumers and firms are likely to react, given changes in their economic environment and the constraints they face. For example, how do different types of consumers respond to advertising or to variation in food availability? To what foods do they substitute when their choice sets are changed or restricted? How do firms react to higher costs due to taxation or to constraints on what they can advertise and sell? Economists have developed a number of sophisticated methods that are well suited to addressing these questions. These include modelling demand in markets for many different products as well as price competition among firms,^50^ flexibly modelling the impact of advertising on consumer demand,^51^ modelling how advertising can influence what products consumers consider purchasing^52^ and modelling pass‐through of cost shocks to consumer prices.^53^ Applying and refining these tools and combining them with the increasingly available micro data on consumer and firm behaviour provides much scope for work that can contribute to policy design. In addition, as different jurisdictions adopt a variety of policies aimed at influencing consumer choice, there are growing opportunities for studies that evaluate the impact of policies already implemented.

In general, policy is interested in affecting consumption and subsequent health outcomes. However, often policy most directly impacts *purchases*, which are an important determinant of, but not necessarily coincident with, consumption. Data on individuals’ consumption (such as those used in this paper) are less well suited to the economic modelling of these policies; they do not, for instance, contain information on purchases, pricing or advertising exposure. Other data sets, including consumer expenditure surveys (such as the Living Costs and Food Survey in the UK and the Consumer Expenditure Survey in the US) and household scanner data (such as the Kantar Worldpanel and Nielsen Homescan), provide detailed information on food and drink purchases, and are useful data sources for those studying policies in this area. The Kantar ‘out‐of‐home’ data are a new source covering individual‐level purchases by UK‐based individuals (aged 13 and above) of all food and drink consumed outside the home. Unlike household‐level purchase data, the out‐of‐home data provide an opportunity to model purchase decisions at the individual level. An important area for future research is to understand the similarities and differences in data that measure food and drink purchases compared with consumption, and to consider how we might combine information from a variety of data sets to learn more about policy design in this area.

## Supporting information

• Appendices A–D

## Footnotes

1 World Health Organisation, 2015.

2 Public Health England, 2017b.

3 Scientific Advisory Committee on Nutrition, 2003.

4 We refer to ‘added sugar’ throughout this paper. In our main data source, we measure this as non‐milk extrinsic sugars. Recommended levels are typically based on ‘free sugars’, which are similar; see Appendix B online for more details. In Section III, we describe the food groups that contribute added sugar to people's diets.

5 See Appendix A online.

6 Some nutritional recommendations are based on the share of total dietary calories from added sugar; the UK recommendation is that added sugar should make up no more than 5 per cent of an individual's caloric intake (as recommended by the Scientific Advisory Committee on Nutrition, SACN). In Appendix B, we show that the patterns described in this paper also hold when added sugar is expressed as a share of calories.

7 World Health Organisation, 2015.

8 See Full Fact (2016).

9 For example, Wang et al. (2012) and Vreman et al. (2017).

10 For a survey, see Rabin (1998).

11 See, for example, O'Donoghue and Rabin (2000).

12 See Bernheim and Rangel (2005).

13 Becker and Murphy (1988) posit a model of rational addiction, in which case the sole regulatory role for government is in correcting for interpersonal externalities.

14 Gruber and Koszegi, 2004.

15 Coase, 1960.

16 Whitman, 2006.

17 For instance, Read and Van Leeuwen (1998) and Gilbert, Gill and Wilson (2002).

18 Cutler, Glaeser and Shapiro, 2003.

19 For instance, see Public Health England (2015) and Centers for Disease Control and Prevention (2016).

20 See, for instance, Baum and Ruhm (2009), Currie (2009), Gortmaker, Long and Wang (2009), Cawley (2010), Currie et al. (2010), Han and Powell (2013) and Azaïs‐Braesco et al. (2017). For more description of consumption patterns, see Rugg‐Gunn et al. (2007) and Ng et al. (2012).

21 Public Health England, 2015; Centers for Disease Control and Prevention, 2016.

22 See, for example, Banerjee and Mullainathan (2010), Mani et al. (2013), Haushofer and Fehr (2014) and Bernheim, Ray and Yeltekin (2015).

23 See Appendix A online.

24 Rennie, Coward and Jebb, 2007; Public Health England, 2016.

25 See, for example, Briefel et al. (1997).

26 Macdiarmid and Blundell, 1998.

27 World Health Organisation, 2007.

28 See, for example, Gruber and Koszegi (2004), O'Donoghue and Rabin (2006) and Haavio and Kotakorpi (2011).

29 See Griffith, O'Connell and Smith (2019).

30 See, for example, Dubois, Griffith and O'Connell (2019).

31 See DellaValle, Roe and Rolls (2005) and Flood, Roe and Rolls (2006).

32 For instance, in a study comparing the Berkeley soda tax with a smaller levy in the state of Washington, Rojas and Wang (2017) find the former was under‐shifted and the latter was over‐shifted. Grogger (2017) finds evidence of over‐shifting of the Mexican soda tax, whereas evidence from the tax in Philadelphia points towards close to complete pass‐through (Seiler, Tuchman and Yao, 2018; Cawley et al., 2018).

33 Grant, 2018; Snowdon, 2018.

34 See, for instance, Allcott, Lockwood and Taubinsky (2019).

35 Tsai, 2016.

36 Bernheim and Rangel, 2005.

37 Livingstone and Helsper, 2006; Bagwell, 2007.

38 Strasburger, 2001.

39 We use advertising data from AC Nielsen and viewing data from the Broadcaster Audience Research Board (BARB) to describe the food and drink advertising that children (aged 4–15) and adults (aged 16–65) saw in 2015. BARB records the number of adult and child impacts that each advert gets. Impacts are a widely used measure of viewing figures, where the number of impacts is equal to the number of pairs of eyes that saw the advert (i.e. five impacts could be five people seeing an advert once or one person seeing the same advert five times). To calculate the average number of adverts per day per child and adult, we divide the total number of impacts by the population of 4‐ to 15‐year‐olds and 16‐ to 65‐year‐olds in 2015 respectively. See Appendix D online for further details on the advertising data.

40 Ofcom, 2016.

41 Adams et al., 2012.

42 Ofcom, 2016.

43 Bagwell, 2007.

44 ASA, 2017.

45 Dubois, Griffith and O'Connell, 2018.

46 Russell, Croker and Viner, 2018.

47 Chiappori, 1992; Chesher, 1998; Cherchye, De Rock and Vermeulen, 2007.

48 See, for example, Goeree (2008) and Eliaz and Spiegler (2011).

49 See Liu et al. (2014).

50 For example, Nevo (2001).

51 For example, Dubois, Griffith and O'Connell (2018).

52 For example, Goeree (2008).

53 For example, Goldberg and Hellerstein (2013).
