# Supplementary material for: What's on the Menu? Policies to Reduce Young People's Sugar Consumption
Source: Fisc Stud. 2020 May 21;41(1):165–97. doi: 10.1111/1475-5890.12194 (PMC7319480; doi:10.1111/1475-5890.12194)
Supplement: Supplementary file 1 — • Appendices A–D [file FISC-41-165-s001.pdf]

Appendices

What's on the menu? Policies to reduce young people's sugar consumption

Rachel Griffith, Martin O'Connell,
Kate Smith and Rebekah Stroud

A National Diet and Nutrition Survey

The National Diet and Nutrition Survey (NDNS) is a survey that provides details of the types and quantities of foods consumed by individuals. We use data from the period covering 2008–16.

Participants are selected by random sample, with oversampling of children relative to adults. We use the sample weights to adjust for differences in sample selection and response (Department of Health (2011)). We observe 12,011 individuals ranging from 1 year old to 96 years old, with children (aged 18 and under) making up just under half of the sample. In our analysis, we split individuals into three-year age bands up until the age of 69, after which we group individuals together in an 'over 70' group. We focus primarily on individuals aged under 21 (6,112 individuals).

The NDNS data describe information from food and drink diaries in which participants record details of their consumption over four consecutive days, including what they ate, portion size and location of consumption. For each individual, we use their mean reported consumption of different food groups and nutrients over the days that they complete the diary. Participants record brand names of food and drink consumed (wherever possible) and collect food label information for any unusual foods in order to help coders identify items and collect nutrient information. For home-made dishes, participants record individual ingredients and quantities for the whole dish as well as information on the cooking method. Using this information, coders identify the nutrient content of each individual's recorded food consumption.

We categorise food and drink into groups defined as in Table A.1.

Table A.1: *Food and drink group definitions*

Food group	Includes	Average share of added sugar (%)
Biscuits & cakes	Biscuits, cakes, puddings and ice cream	18.9
Confectionery	Confectionery	12.7
Jams & spreads	Jams, breakfast spreads, butters and fats	10.5
Cereal	All cereal	6.8
Dairy	Cheese and yogurt	4.8
Other food	Meat, fish, crisps, fruit, vegetables, pasta, rice, bread, potatoes, eggs, sauces, toddler food and other miscellaneous	9.6
**Drink group**	**Includes**	**Average share of added sugar (%)**
Soft drinks	Soft drinks, both diet and non-diet	22.2
Fruit juice	Fruit jucie	11.2
Alcohol	Alcohol	1.5
Other	Milk and hot drinks	1.8

Note: This table lists all food and drink products included in each food and drink group. The last column shows the average share of added sugar that young people get from each of these food groups, as recorded in NDNS.

B Definition and recommended maximum levels of added sugar

Definition of added sugar

In this paper, we use 'added sugar' to refer to non-milk extrinsic sugars, as recorded in the National Diet and Nutrition Survey. 'Non-milk extrinsic sugars' includes all sugar captured by the definition of 'free sugars' and also includes half of the sugars present in dried, canned or stewed fruit. 'Free sugars' refers to monosaccharides (single sugar units, e.g. glucose and fructose) and disaccharides (two single units joined together, e.g. sucrose) that are added to foods and drinks by the manufacturer, cook or consumer, as well as sugars naturally present in honey, syrups, fruit juices and fruit juice concentrates.

Recommended maximum daily intake of added sugar

The NHS's online Eat Well guide (NHS (2017)) recommends the following:

- Adults should have no more than 30g of added sugar a day.
- Children aged 7–10 should have no more than 24g of added sugar a day.
- Children aged 4–6 should have no more than 19g of added sugar a day.
- There is no guideline limit for children under the age of 4, but it is recommended that they avoid all food and drink containing added sugar.

Other advice differs, in terms of both the magnitude of recommendations and their form. For example, the World Health Organisation (WHO) and the Scientific Advisory Committee on Nutrition (SACN) both formulate their recommendations in terms of the share of calories from added sugar. WHO recommends that added sugar consumption should not exceed 10 per cent of total calorie intake, whereas SACN recommends that consumption should not exceed 5 per cent of total calorie intake. Figure B.1 shows how individuals' added sugar share of calories varies with age and compares consumption with SACN's recommendations. We see that, although the precise recommendations vary, sugar consumption far exceeds each set of recommendations and this is particularly true for young people. In Section B.1, we reproduce the figures from Section III in terms of the share of calories that young people get from added sugar and show that the same patterns of young people's sugar consumption are still observed.

Figure B.1: *Share of calories from added sugar by age*

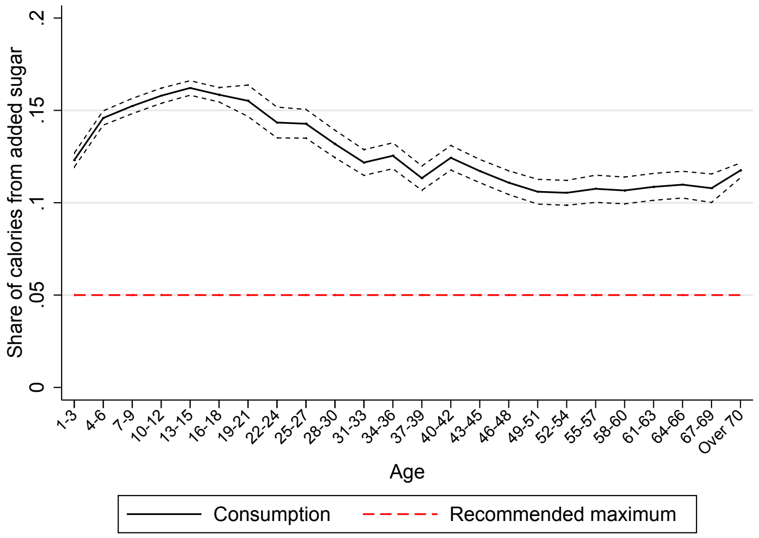

Note: The solid line shows the average share of calories from added sugar of individuals in each three-year age band, based on the National Diet and Nutrition Survey (see Appendix A). Added sugar refers to non-milk extrinsic sugars, as recorded in the NDNS. The dotted lines show the 95% confidence intervals. The dashed red line shows the daily maximum recommended share of calories from added sugar, as recommended by the Scientific Advisory Committee on Nutrition.

It should also be noted that recommendations are typically stated in terms of free sugars whereas our analysis is in terms of non-milk extrinsic sugars. Non-milk extrinsic sugars and free sugars are equivalent with the exception of products containing dried, canned or stewed fruit. These products make up only a small part of overall non-milk extrinsic sugar consumption, so this measurement error is likely to be small.

B.1 Describing young people's added sugar consumption as a share of calorie intake

Distribution of added sugar consumption

Figure B.2: *Distribution of share of calories from added sugar by age*

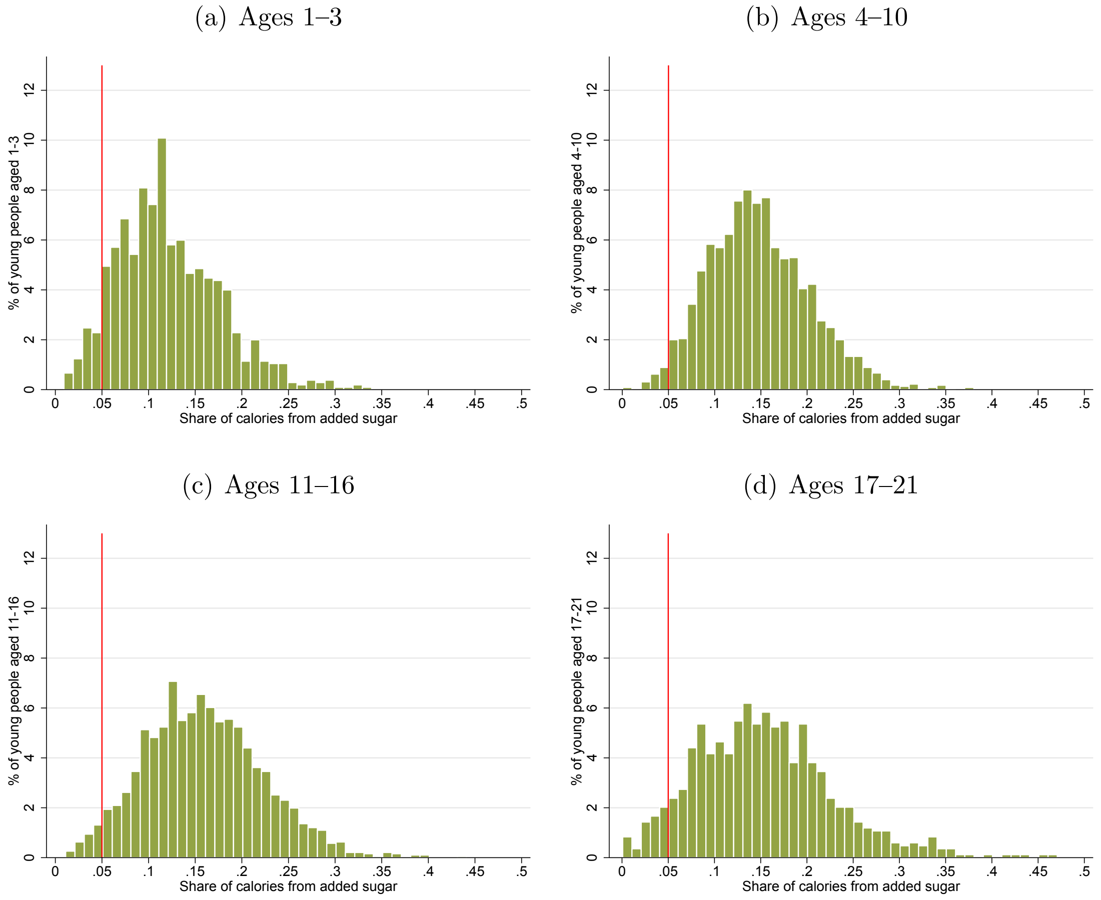

Note: The graphs show the distribution of the share of calories got from added sugar for young people aged (a) 1–3, (b) 4–10, (c) 11–16 and (d) 17–21. Each figure is trimmed at the 99th percentile of the distribution for each age group. Bin width is 0.01. The vertical red lines show the recommended maximum daily share of calories from added sugar for young people, as recommended by SACN (5%). Data from the NDNS; the sample comprises 6,112 individuals aged 1–21 over 2008–16. See Appendix A for more details on the data.

Sources of added sugar consumption

Figure B.3: *Share of calories from added sugar from food and drink, by age*

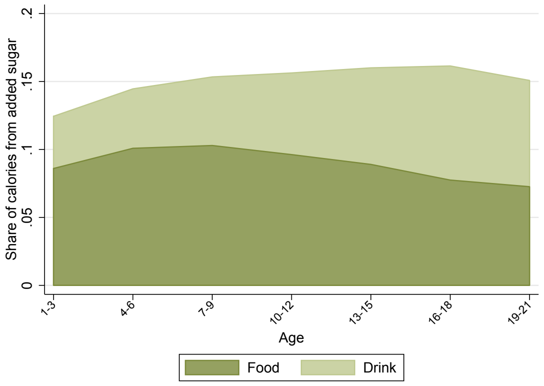

Note: The darker green area shows the average share of calories from added sugar that individuals in each three-year age band get from food, while the lighter green area shows the average share of calories from added sugar that they get from drink. Data from the NDNS; the sample comprises 6,112 individuals aged 1–21 over 2008–16. See Appendix A for more details on the data.

Figure B.4: *Share of calories from added sugar by type of food and drink and by age*

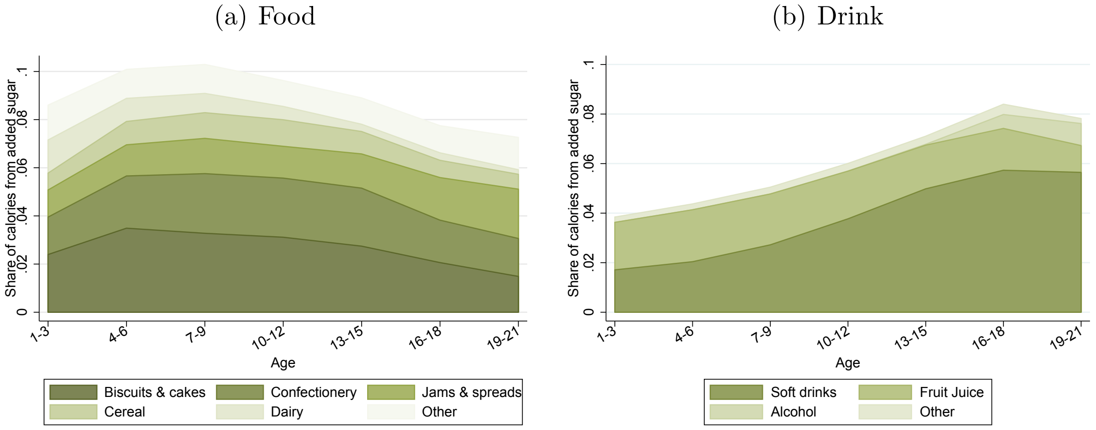

Note: The graphs show the average share of calories from added sugar that individuals within each three-year age band obtain from each food group (left-hand panel) and each drink group (right-hand panel). Data are from the NDNS; sample comprises 6,112 individuals aged 1–21 over 2008–16. Groups are ordered (from bottom to top) in terms of the average amount of added sugar that individuals under 21 years old get from that food or drink group (with the exception of 'other', which is placed at the top). See Appendix A for more details on the data, including food and drink group definitions.

Table B.1: *Share of added sugar from soft drinks and confectionery*

Share of calories from added sugar (%)	% of added sugar from Soft drinks	Confectionery	Biscuits and cakes
< 5	8.2	7.6	20.6
5-10	13.8	9.6	22.0
10-15	17.4	12.4	21.2
> 15	29.9	14.5	15.9

Note: This table shows the share of added sugar that individuals get from soft drinks, from confectionery and from biscuits and cakes by the sugar intensity of their diet – i.e. the share of calories they get from added sugar. Data are from the NDNS; sample comprises 6,112 individuals aged 1–21 over 2008–16. See Appendix A for more details on the data.

Figure B.5: *Distribution of share of calories from added sugar including and excluding consumption of soft drinks and confectionery*

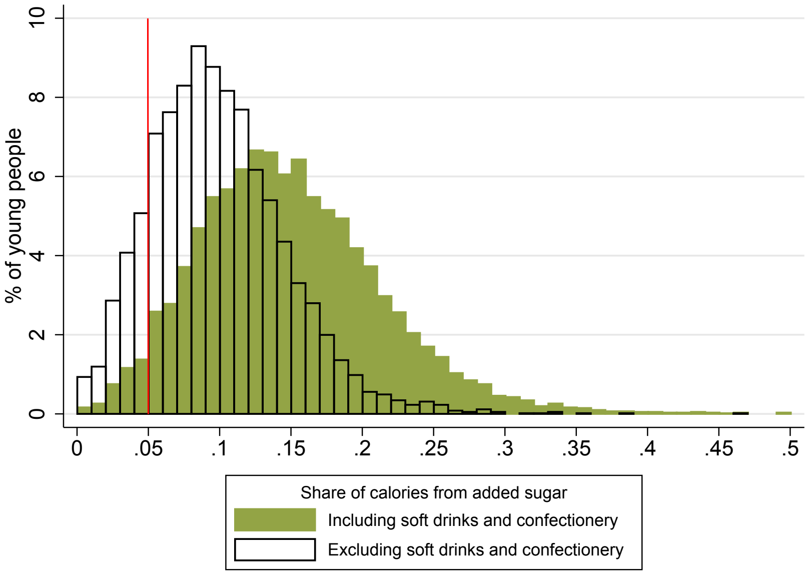

Note: The graph shows the distribution of young people's share of calories from added sugar (green bars) and their share of calories from added sugar when excluding consumption of soft drinks and confectionery (bars with black outline). The vertical red line shows the recommended maximum daily share of calories from added sugar for young people, as recommended by SACN (5%). Bin width is 0.01. Data are from the NDNS; sample comprises 6,112 individuals aged 1–21 over 2008–16. See Appendix A for more details on the data.

Who buys the sugar that young people eat?

Figure B.6: *Share of calories from added sugar by location and age*

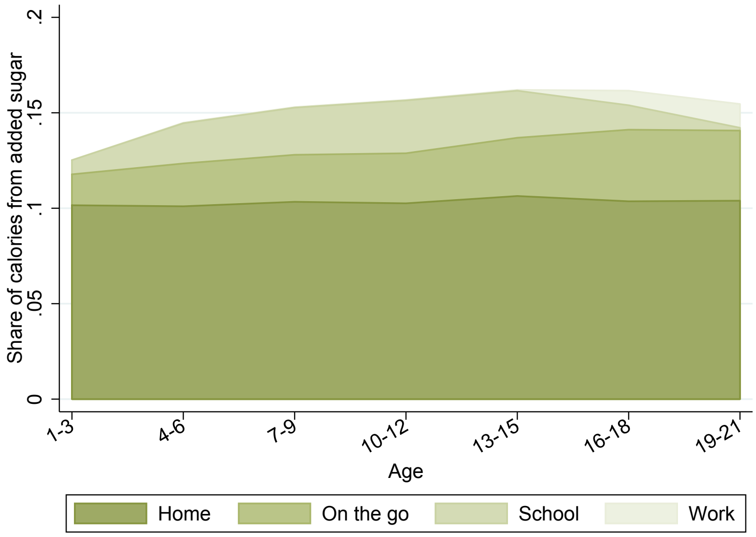

Note: The graph shows the average share of calories from added sugar that individuals consume at home, on the go, at school or at work, as recorded in the NDNS. Sample is 6,112 individuals aged 1–21 over 2008–16. Locations are ordered (from bottom to top) in terms of the average amount of added sugar individuals under 21 years old consume in that location. See Appendix A for more details on the data.

Figure B.7: *Distribution of share of calories from added sugar: all consumption and at-home consumption only*

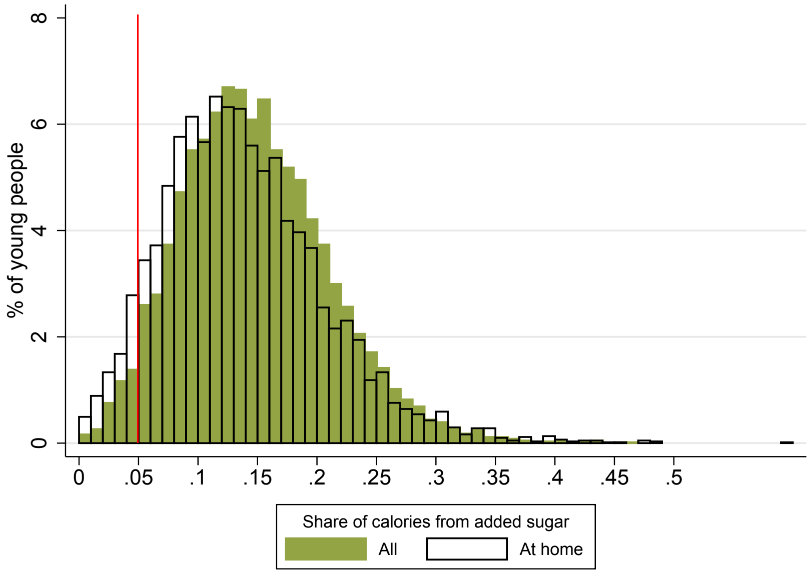

Note: The graph shows the distribution of young people's share of calories from added sugar (green bars) and share of calories from added sugar when considering at-home consumption only (bars with black outline). The vertical red line shows the recommended maximum daily share of calories from added sugar for young people, as recommended by SACN (5%). Bin width is 0.01. Data are from the NDNS; sample comprises 6,112 individuals aged 1–21 over 2008–16. See Appendix A for more details on the data.

Figure B.8: *Share of calories from added sugar from food and drink by location and age*

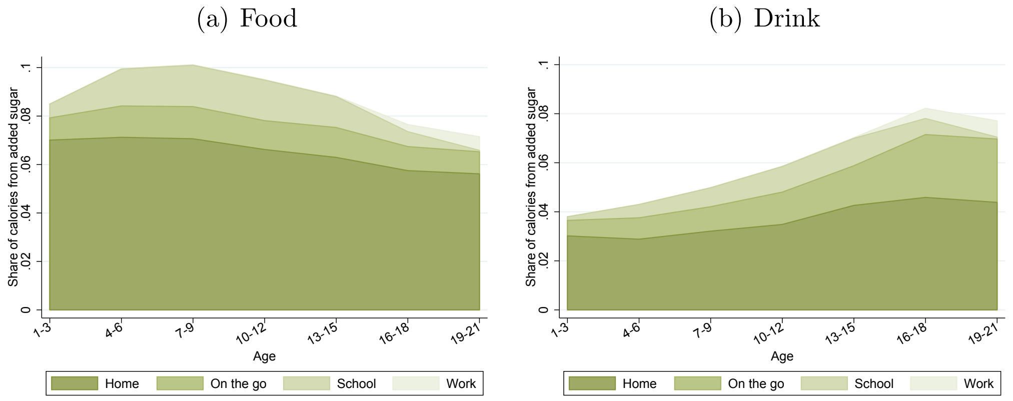

Note: These graphs show the average share of calories from added sugar that individuals from each three-year age band get from food (left-hand panel) and from drink (right-hand panel) that is consumed at home, on the go, at school and at work. Locations are ordered (from bottom to top) in terms of the average amount of added sugar that individuals aged under 21 years consume in that location. Data are from the NDNS; sample comprises 6,112 individuals aged 1–21 over 2008–16. See Appendix A for more details on the data.

Table B.2: *Share of added sugar that is consumed out of the home*

Share of calories from added sugar (%)	% of added sugar from *food and drink* consumed out of the home	% of added sugar from *soft drinks* consumed out of the home	% of added sugar from *confectionery* consumed out of the home
< 5	21.3	30.3	19.0
5-10	26.8	34.9	32.2
10-15	31.5	38.6	39.6
> 15	33.5	43.2	41.1

Note: This table shows the share of added sugar that young people get from all food and drink, from soft drinks and from confectionery that they consume out of the home, by sugar intensity of diet – i.e. the share of calories they get from added sugar. Data are from the NDNS; sample comprises 6,112 individuals aged 1–21 over 2008–16. See Appendix A for more details on the data.

C Over-19-year-olds' added sugar consumption

Figure C.1: *Distribution of added sugar consumption by age*

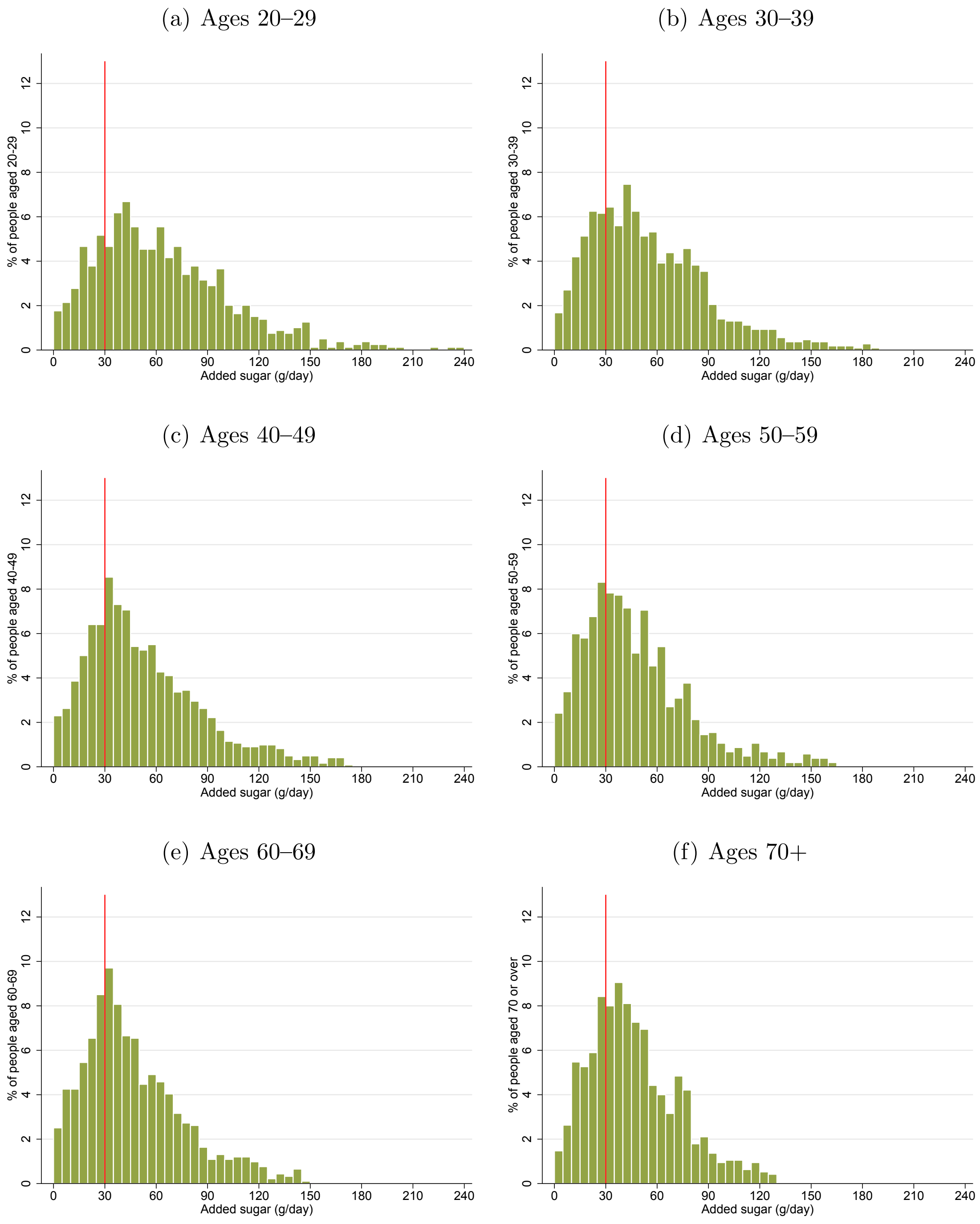

Note: The graphs show the distribution of added sugar consumption (grams per day) for people aged (a) 20–29, (b) 30–39, (c) 40–49, (d) 50–59, (e) 60–69 and (f) 70 and over. Each figure is trimmed at the 99th percentile of the distribution for each age group. Bin width is 5g. The vertical red lines represent the recommended maximum daily added sugar consumption (30g). Data from the NDNS; the sample covers the period 2008–16 and comprises 802 individuals aged 20-29, 1,083 aged 30-39, 1,230 aged 40-49, 1,046 aged 50-59, 927 aged 60-69 and 959 aged 70 or over. See Appendix A for more details on the data.

D Advertising data

We use data from Nielsen on all adverts for food and drink products broadcast on television during 2015. The data include information on the brand advertised, the channel on which the advert was shown, what programmes ran either side of the advert and the numbers of child and adult impacts that the advert received (a widely used measure of viewing figures) provided by the Broadcasters' Audience Research Board (BARB).

The average number of adverts seen a day by each child and adult in the UK are calculated by dividing the number of child and adult impacts by the size of the child and adult populations in 2015 respectively (9,054,191 children aged 4–15; 41,941,834 adults aged 16–65). Impacts measure the number of pairs of eyes that saw the advert (i.e. five impacts could be five people seeing an advert once or one person seeing the same advert five times).

HFSS definition

In order to distinguish between advertising that is HFSS and advertising that is non-HFSS, we construct a nutrient profile score for each brand in the Nielsen data. This follows the same approach as in Griffith et al. (2018); for more details, see appendix B of that report.

For each brand that is recorded as being advertised in the Nielsen data, we match the nutrient information on the products that comprise each brand from the Kantar Worldpanel (supplementing with information from other sources when necessary). Note that it is often the case that the brand-level information in the Nielsen data aggregates over many products in the Kantar data (either because many products are advertised or because it is the wider brand that is being promoted); we describe below how we treat these brands.

We apply the Nutritional Profiling Model (NPM) to these data to calculate an NPM score for each brand advertised (Rayner et al. (2005)). A product is defined as HFSS if it has an NPM score above or equal to 4 for foods and 1 for drinks. If all products comprising the brand recorded in the advertising data have an NPM score above (below) the threshold, we classify them as HFSS (non-HFSS). For brands that comprise some products above and some below the threshold, we apportion the impacts for these adverts in proportion to the share of transactions (of the brand) made up by products above and below. For example, if the brand 'Coca Cola' is listed as being advertised, and 40 per cent of transactions of Coca Cola are for Coke Regular (which is above the threshold) and 60 per cent are for Diet Coke

(which is below the threshold), then 40 per cent of the impacts for these adverts would be classed as HFSS and 60 per cent as non-HFSS.

References

Department of Health (2011). National Diet and Nutrition Survey Headline results from Years 1 and 2 (combined) of the Rolling Programme. Appendix B: Weighting the NDNS core sample. pp. 34.

Griffith, R., M. O'Connell, K. Smith, and R. Stroud (2018). Children's exposure to TV advertising of food and drink. *IFS Briefing Note BN238*, 15.

NHS (2017). How does sugar in our diet affect our health? - NHS. https://www.nhs.uk/live-well/eat-well/how-does-sugar-in-our-diet-affect-our-health/.

Rayner, M., P. Scarborough, A. Boxer, and L. Stockley (2005). *Nutrient Profiles: Development of Final Model. Final Report for the Food Standards Agency.* Oxford: British Heart Foundation Health Promotion Research Group, Department of Public Health, University of Oxford.
